# Heterologous prime-boost with A(H5N1) pandemic influenza vaccines induces broader cross-clade antibody responses than homologous prime-boost

**DOI:** 10.1038/s41541-019-0114-8

**Published:** 2019-05-29

**Authors:** Min Z. Levine, Crystal Holiday, Stacie Jefferson, F. Liaini Gross, Feng Liu, Sheng Li, Damien Friel, Philippe Boutet, Bruce L. Innis, Corey P. Mallett, Terrence M. Tumpey, James Stevens, Jacqueline M. Katz

**Affiliations:** 1 Influenza Division, Centers for Disease Control and Prevention, Atlanta, GA USA; 2 Battelle Memorial Institute, Atlanta, GA USA; 3 GSK Vaccines, Wavre, Belgium; 4 GSK Vaccines, Rockville, MD USA; 5 Biomedical Advanced Research and Development Authority, Washington, DC USA; 6 Present Address: Sciogen, Los Altos, CA USA; 7 Present Address: PATH, Washington, DC USA

**Keywords:** Immunology, Diseases, Medical research, Influenza virus

## Abstract

Highly pathogenic avian influenza (HPAI) A(H5Nx) viruses continue to pose a pandemic threat. US national vaccine stockpiles are a cornerstone of the influenza pandemic preparedness plans. However, continual genetic and antigenic divergence of A(H5Nx) viruses requires the development of effective vaccination strategies using stockpiled vaccines and adjuvants for pandemic preparedness. Human sera collected from healthy adults who received either homologous (2 doses of a AS03_A_-adjuvanted A/turkey/Turkey/1/2005, A/Turkey), or heterologous (primed with AS03_A_-adjuvanted A/Indonesia/5/2005, A/Indo, followed by A/Turkey boost) prime-boost vaccination regimens were analyzed by hemagglutination inhibition and microneutralization assays against 8 wild-type HPAI A(H5Nx) viruses from 6 genetic clades. Molecular, structural and antigenic features of the A(H5Nx) viruses that could influence the cross-clade antibody responses were also explored. Compared with homologous prime-boost vaccinations, priming with a clade 2.1.3.2 antigen (A/Indo) followed by one booster dose of a clade 2.2.1 antigen (A/Turkey) administered 18 months apart did not compromise the antibody responses to the booster vaccine (A/Turkey), it also broadened the cross-clade antibody responses to several antigenically drifted variants from 6 heterologous clades, including an antigenically distant A(H5N8) virus (A/gyrfalcon/Washington/410886/2014, clade 2.3.4.4) that caused recent outbreaks in US poultry. The magnitude and breadth of the cross-clade antibody responses against emerging HPAI A(H5Nx) viruses are associated with genetic, structural and antigenic differences from the vaccine viruses and enhanced by the inclusion of an adjuvant. Heterologous prime-boost vaccination with AS03_A_ adjuvanted vaccine offers a vaccination strategy to use existing stockpiled vaccines for pandemic preparedness against new emerging HPAI A(H5Nx) viruses.

## Influenza vaccines: Heterologous prime-boost broadens cross-clade responses

Influenza viruses are highly variable and display continuous antigenic drift, limiting the effectiveness of vaccine stockpiles and demanding new strategies to enhance vaccine effectiveness. Here, Min Levine from the Centers for Disease Control and Prevention and colleagues report a heterologous prime-boost A(H5N1) vaccination regimen that induced a broader cross-clade response when compared with homologous vaccination. In the study, adults primed with a clade 2.1.3.2 antigen (A/Indo) followed by one booster dose of a clade 2.2.1 antigen (A/Turkey) presented with enhanced hemagglutinin inhibition and neutralizing antibody titers to eight A(H5Nx) viruses without limiting the antibody response to the A/Turkey booster vaccine. Given that no individual H5 clade has led to protection against all H5 viruses, heterologous vaccination strategies that provide cross-clade reactivity may lead to more effective protection against influenza virus infection.

## Introduction

As we commemorate the 100 years since the 1918 influenza pandemic, the threat of a potential new pandemic remains with influenza viruses undergoing antigenic drift and shift. Since the identification of human infections with the highly pathogenic avian influenza (HPAI) A(H5N1) viruses over two decades ago, these viruses have become endemic in poultry and wild birds in many countries. They continue to cause sporadic human infections with high disease severity and high mortality (≥50% fatality). By December 2018, the World Health Organization (WHO) reported 860 cases of human infections with A(H5N1) viruses since 2003.^1^ A(H5N1) HPAI viruses display remarkable genetic and antigenic divergence, with very little to no immunity exists in the human population, highlighting the pandemic potential of these viruses.^2^ To date, A (H5N1) viruses have evolved into 10 phylogenetically distinct first order HA clades and over 40 subclades.^3^ Viruses from clades 0, 1, 2, and 7 have caused human disease,^4–6^ but clade 1 and clade 2 viruses are responsible for most of the human infections.^4^ Recently, A(H5N1) viruses derived from A/goose/Guangdong/1/1996 HA lineage have also reassorted with other avian influenza viruses, acquiring multiple neuraminidases (NA) subtypes, designated as A(H5Nx) viruses.^3^ Although in recent years the number of A(H5N1) human infections has decreased, sporadic A(H5Nx) cases continue to occur.^7^

Vaccination is a critical component of public health measures to protect the population against influenza infections. Previous studies have demonstrated that two doses of adjuvanted A(H5N1) vaccines administered as a prime-boost regimen are needed to elicit sufficient levels of antibody responses thought to confer protection.^8,9^ In the United States, development of national vaccine stockpiles is a cornerstone of the pandemic preparedness plans.^10^ In addition, candidate vaccine viruses (CVVs) are developed to match emerging strains. However, rapid evolution of the A(H5Nx) viruses requires constant updating of such CVVs. In the face of a pandemic, the likelihood of antigenic mismatch between the pandemic strain and the existing stockpiled vaccine strains is fairly high. Even when the CVV to the pandemic strain becomes available, the existing vaccine manufacturing capacity may not be sufficient to provide enough quantities of matched vaccines rapidly to protect the entire population against the pandemic virus. Thus, improved influenza A(H5Nx) vaccination strategies utilizing existing stockpiled vaccines that can induce broader cross-reactive neutralizing antibody responses to emerging A(H5Nx) pandemic strains are highly desirable.

Recently, we demonstrated that heterologous boost vaccination with MF59-adjuvanted clade 2.3.4 A(H5N1) virus following a non-adjuvanted clade 1 virus prime can elicit modest cross-clade neutralizing antibodies against newly emerged A(H5Nx) viruses from clade 2.3.4.4.^11^ In the current study, we evaluated the magnitude and the breadth of the cross-clade antibody responses following either homologous or heterologous prime-boost vaccination in adults against wild type A(H5Nx) HPAI viruses from 6 genetic clades that caused either human diseases or animal outbreaks in the recent years. We chose antigens and adjuvants that are currently a part of the US National Pre-pandemic Influenza Vaccine Stockpile: homologous vaccination with 2 doses of a AS03_A_-adjuvanted clade 2.2.1 virus (A/turkey/Turkey/1/2005, A/Turkey), versus heterologous vaccination primed with an AS03_A_-adjuvanted clade 2.1.3.2 virus (A/Indonesia/5/2005, A/Indo) followed by a single booster dose with AS03_A_-adjuvanted A/Turkey, 18 months apart. We also explored the genetic, structural and antigenic characteristics of the A(H5Nx) viruses that may influence the levels of cross-clade antibody responses induced by the prime-boost vaccinations.

## Results

### Increased antigenic diversity of A(H5Nx) viruses pose challenges to the selection of stockpiled vaccines

HPAI A(H5N1) and A(H5N8) viruses from genetic clades that caused human infections and animal outbreaks in recent years were selected to evaluate the breadth of the antibody responses following either homologous or heterologous prime-boost vaccination with A(H5N1) vaccines. A(H5Nx) viruses have diverged into multiple phylogenetic clades; 8 viruses from 6 genetic clades were included in the study (Supplementary Fig. 1). As characterized by primary infection ferret antisera (Table 1), recent circulating A(H5Nx) viruses demonstrated increased antigenic diversity as compared to A/Turkey and A/Indo, two of the currently stockpiled vaccines based on older viruses that emerged over a decade ago. Ferret antisera generated against vaccine viruses A/Turkey and A/Indo exhibited ≥4 fold reduction against most of the contemporary viruses from heterologous clades compared to the titers of the homologous vaccine viruses, by both HI and MN (Table 1). Among all the viruses evaluated, the recently emerged A(H5N8) virus from clade 2.3.4.4 (A/gyrfalcon/Washington/410886/2014, A/GF/14) that caused multiple outbreaks in US poultry since late 2014,^12^ had ≥128-fold two-way reduction by HI when compared to both vaccine viruses (Table 1), suggesting the A(H5N8) virus is antigenically quite distant from the two stockpiled vaccines. Moreover, characterization by ferret antisera demonstrated that viruses from each of the genetic clades are antigenically distinct from those in heterologous clades, highlighting the challenges to select pandemic stockpiled vaccines based on single vaccine antigens.

**Table 1:** Antigenic characterization of A(H5Nx) viruses using ferret sera by HI and MN

Assays	Influenza A viruses	HA clades	Ferret Antisera							
			A/Vietnam/1194/2004	A/Indonesia/5/2005	A/Indonesia/NIHRD-12379/2012	A/turkey/Turkey/1/2005	A/Egypt/01050/NAMRU/2013	A/duck/Bangladesh/19097/2013	A/duck/Vietnam/NCVD-1584/2012	A/gyrfalcon/WA/410886/2014
HI	A/Vietnam/1194/2004	1	1280	320	160	320	160	80	160	10
	A/Indonesia/5/2005^a^	2.1.3.2	160	1280	320	160	320	320	320	10
	A/Indonesia/NIHRD-12379/2012	2.1.3.2	160	320	1280	80	40	40	160	10
	A/turkey/Turkey/1/2005^a^	2.2.1	160	640	160	2560	640	640	1280	10
	A/Egypt/N04915/2014	2.2.1	160	320	113	640	2560	320	320	320
	A/duck/Bangladesh/19097/2013	2.3.2.1a	160	320	160	640	226	1280	2560	10
	A/duck/Vietnam/NCVD-1584/2012	2.3.2.1c	80	320	80	640	320	640	2560	10
	A/gyrfalcon/Washington/410886/2014	2.3.4.4	10	10	40	10	1280	10	10	2560
MN	A/Vietnam/1194/2004	1	640	40	20	40	20	20	20	20
	A/Indonesia/5/2005^a^	2.1.3.2	160	1280	113	57	160	57	40	20
	A/Indonesia/NIHRD-12379/2012	2.1.3.2	160	320	1280	57	20	20	80	20
	A/turkey/Turkey/1/2005^a^	2.2.1	160	640	113	2560	1280	640	1280	20
	A/Egypt/N04915/2014	2.2.1	80	57	80	640	5120	160	320	640
	A/duck/Bangladesh/19097/2013	2.3.2.1a	40	80	40	160	28	640	1280	20
	A/duck/Vietnam/NCVD-1584/2012	2.3.2.1c	20	57	20	226	40	320	1280	20
	A/gyrfalcon/Washington/410886/2014	2.3.4.4	20	20	40	40	5120	40	40	5120

A(H5Nx) viruses were tested against ferret antisera by HI and MN. Titers to ferret antisera generated against homologous viruses are underlined

^a^Vaccine components

### HI and neutralizing antibody responses following homologous and heterologous vaccination in adults

All participants received 2 doses of AS03_A_-adjuvanted A(H5N1) vaccines (Table 2). In the heterologous prime-boost groups, the booster dose was administered around 18 months following the initial priming with A/Indo. Day 559 (day 10 post-boost) represents the peak of the antibody responses from all the time points at which sera were collected in the initial immunogenicity evaluation of this clinical trial,^13^ thus were included in the current study to assess the breadth of the cross-clade antibody responses.

**Table 2:** Homologous and heterologous A(H5N1) vaccine groups assessed

Study groups	Vaccine groups	Prime		Boost
		D0	6 months (day 182)	18 months (day 549)
1	Homologous	Placebo (PBS)	Turkey 3.75 µg + AS03_A_	Turkey 3.75 µg + AS03_A_
2	Heterologous(high dose)	Indo 7.5 µg + AS03_A_	Placebo (PBS)	Turkey 7.5 µg + AS03_A_
3	Heterologous(low dose)	Indo 3.75 µg + AS03_A_	Placebo (PBS)	Turkey 3.75 µg + AS03_A_

The majority of the participants were serologically naive to A(H5Nx) influenza viruses before receiving A(H5N1) vaccination with baseline titers below the limit of detection to almost all 8 A(H5Nx) viruses (GMTs: 5–8, 95% CI 5–10 by both HI and MN). Following vaccination, participants in heterologous prime-boost groups (groups 2 & 3) that only received one dose of A/Turkey post A/Indo prime had higher HI and MN antibody titers to the vaccine virus A/Turkey than those in the homologous prime-boost group (group 1) that received 2 doses of A/Turkey (Tables 3, 4).

**Table 3:** Cross-clade HI antibody responses following prime-boost A(H5N1) vaccination

Study groups	Influenza A viruses	Subtype	HA clade	*N*	Pre-vaccination HI GMT (95% CI)	Post-vaccination HI GMT 95% CI	%with HI ≥40 post vaccination- SPR (95% CI)
Group 1 (homologous)	A/turkey/Turkey/1/2005	H5N1	2.2.1	35	5 (–)	122 (77–194)	86 (70–95)
	A/Indonesia/5/2005	H5N1	2.1.3.2	35	5 (–)	55 (34–88)	69 (51–83)
	A/Vietnam/1194/2004	H5N1	1	35	6 (5–7)	55 (36–84)	71 (54–85)
	A/Indonesia/NIHRD-12379/2012	H5N1	2.1.3.2	35	5 (5–6)	40 (26–62)	63 (45–79)
	A/Egypt/N04915/2014	H5N1	2.2.1	35	5 (5–6)	43 (27–68)	66 (48–81)
	A/duck/Bangladesh/19097/2013	H5N1	2.3.2.1a	35	5 (5–6)	42(27–66)	66 (48–81)
	A/duck/Vietnam/NCVD-1584/2012	H5N1	2.3.2.1c	35	5 (–)	36 (22–56)	57 (39–74)
	A/gyrfalcon/Washington/410886/2014	H5N8	2.3.4.4	35	5 (–)	15 (10–22)	26 (12–43)
	A/turkey/Turkey/1/2005	H5N1	2.2.1	35	5 (–)	148 (94–234)	83 (66–93)
Group 2 (heterologous-high dose)	A/Indonesia/5/2005	H5N1	2.1.3.2	35	5 (5–6)	145 (93–226)	83 (66–93)
	A/Vietnam/1194/2004	H5N1	1	35	5 (5–6)	61 (41–89)	77 (60–90)
	A/Indonesia/NIHRD-12379/2012	H5N1	2.1.3.2	35	5 (5–6)	109 (69–171)	83 (66–93)
	A/Egypt/N04915/2014	H5N1	2.2.1	35	6 (5–7)	53 (36–76)	71 (54–85)
	A/duck/Bangladesh/19097/2013	H5N1	2.3.2.1a	35	5 (5–6)	61 (42–89)	80 (63–92)
	A/duck/Vietnam/NCVD-1584/2012	H5N1	2.3.2.1c	35	5 (5–6)	56 (37–84)	74 (57–88)
	A/gyrfalcon/Washington/410886/2014	H5N8	2.3.4.4	35	5 (–)	23(16–32)	40 (24–58)
	A/turkey/Turkey/1/2005	H5N1	2.2.1	35	6 (5–6)	168 (110–257)	94 (81–99)
Group 3 (heterologous-low dose)	A/Indonesia/5/2005	H5N1	2.1.3.2	35	5 (–)	155 (99–243)	91 (77–98)
	A/Vietnam/1194/2004	H5N1	1	35	6 (5–7)	75 (50–115)	80 (63–92)
	A/Indonesia/NIHRD-12379/2012	H5N1	2.1.3.2	35	5 (–)	119 (77–184)	89 (73–97)
	A/Egypt/N04915/2014	H5N1	2.2.1	35	5 (5–6)	51 (33–77)	71 (54–85)
	A/duck/Bangladesh/19097/2013	H5N1	2.3.2.1a	35	5 (–)	68 (46–102)	74 (57–88)
	A/duck/Vietnam/NCVD-1584/2012	H5N1	2.3.2.1c	35	5 (–)	64 (41–100)	69 (51–83)
	A/gyrfalcon/Washington/410886/2014	H5N8	2.3.4.4	35	5 (–)	24 (15–37)	34 (19–52)

*CI* confidence interval

**Table 4:** Cross-clade neutralizing antibody responses following prime-boost vaccination

Study groups	Influenza A viruses	Subtype	HA clade	*N*	Pre-vaccination MN GMT (95% CI)	Post-vaccination MN GMT (95% CI)	%with MN ≥40 post vaccination—SPR (95% CI)
	A/turkey/Turkey/1/2005	H5N1	2.2.1	35	6 (5–7)	466 (300–725)	94 (81–99)
Group 1 (homologous)	A/Indonesia/5/2005	H5N1	2.1.3.2	35	5 (-)	92 (56–150)	80 (63–92)
	A/Vietnam/1194/2004	H5N1	1	35	5 (5–6)	47 (31–71)	63 (45–79)
	A/Indonesia/NIHRD-12379/2012	H5N1	2.1.3.2	35	5 (5–6)	62 (40–98)	63 (45–79)
	A/Egypt/N04915/2014	H5N1	2.2.1	35	7 (6–8)	156 (98–249)	89 (73–97)
	A/duck/Bangladesh/19097/2013	H5N1	2.3.2.1a	35	7 (5–8)	80 (48–133)	77 (60–90)
	A/duck/Vietnam/NCVD-1584/2012	H5N1	2.3.2.1c	35	5 (–)	54 (32–92)	57 (39–74)
	A/gyrfalcon/Washington/410886/2014	H5N8	2.3.4.4	35	7 (6–9)	57 (33–99)	63 (45–79)
	A/turkey/Turkey/1/2005	H5N1	2.2.1	35	6 (5–7)	526 (341–810)	97 (85–100)
Group 2 (heterlogous high dose)	A/Indonesia/5/2005	H5N1	2.1.3.2	35	5 (5–6)	284 (176–459)	91 (77–98)
	A/Vietnam/1194/2004	H5N1	1	35	5 (–)	60 (39–94)	69 (51–83)
	A/Indonesia/NIHRD-12379/2012	H5N1	2.1.3.2	35	6 (5–7)	158 (98–254)	83 (66–93)
	A/Egypt/N04915/2014	H5N1	2.2.1	35	8 (6–10)	188 (128–277)	89 73–97)
	A/duck/Bangladesh/19097/2013	H5N1	2.3.2.1a	35	6 (5–7)	100 (62–159)	80 (63–92)
	A/duck/Vietnam/NCVD-1584/2012	H5N1	2.3.2.1c	35	5 (–)	73 (45–118)	74 (57–88)
	A/gyrfalcon/Washington/410886/2014	H5N8	2.3.4.4	35	7 (5–8)	49 (29–81)	63 (45–79)
	A/turkey/Turkey/1/2005	H5N1	2.2.1	35	6 (5–8)	643 (465–889)	100 (90–100)
Group 3 (heterologous-low dose)	A/Indonesia/5/2005	H5N1	2.1.3.2	35	5 (–)	333 (218–508)	91 (77–98)
	A/Vietnam/1194/2004	H5N1	1	35	5 (5–6)	70 (44–110)	77 (60–90)
	A/Indonesia/NIHRD-12379/2012	H5N1	2.1.3.2	35	5 (–)	180 (114–283)	89 (73–97)
	A/Egypt/N04915/2014	H5N1	2.2.1	35	6 (5–7)	181 (116–284)	86 (70–95)
	A/duck/Bangladesh/19097/2013	H5N1	2.3.2.1a	35	6 (5–7)	96 (56–163)	83 (66–93)
	A/duck/Vietnam/NCVD-1584/2012	H5N1	2.3.2.1c	35	5 (–)	66 (40–111)	74 (57–88)
	A/gyrfalcon/Washington/410886/2014	H5N8	2.3.4.4	35	6 (5–7)	61 (36–101)	74 (57–88)

*CI* confidence interval

**Fig. 1:**
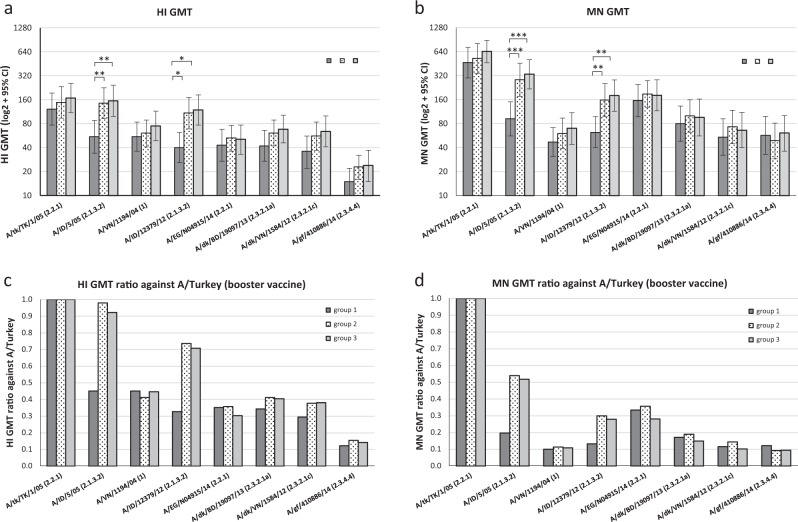
HI and MN antibody responses post homologous and heterologous prime-boost vaccination. **a** Geometric mean titers (GMTs) of the HI antibody responses from each of the 3 study groups post vaccination, **b** GMTs of neutralizing antibody responses of each of the 3 study groups post vaccination. **c** HI GMT ratios against A/Turkey; **d** MN GMT ratios against A/Turkey. Error bars indicate 95% confidence intervals (CI) of HI and MN responses from each group (*N* = 35 per group). Groups that achieved statistical difference in responses were indicated by: *p < 0.05, **p < 0.01, ***p < 0.001. A/tk/TK/05 (A/turkey/Turkey/1/2005), A/ID/5/05 (A/Indonesia/5/2005), A/VN/1194/04/(A/Vietnam/1194/2004), A/ID/12379/12 (A/Indonesia/NIHRD-12379/2012), A/EG/N04915/14 (A/Egypt/N04915/2014), A/dk/BD/19097/13 (A/duck/Bangladesh/19097/2013), A/dk/VN/1584/12 (A/duck/Vietnam/NCVD-1584/2012), A/gf/410886/14 (A/gyrfalcon/Washington/410886/2014)

Compared with the homologous prime-boost group (group 1), subjects in the heterologous prime-boost groups (groups 2 & 3) also mounted stronger and broader cross-clade HI and MN antibody responses to antigenically diverse variants. HI (Table 3; Fig. 1a) and neutralizing antibody titers (Table 4; Fig. 1b) to all 8 viruses tested were higher in the heterologous prime-boost groups than those in the homologous prime-boost group. HI and neutralizing antibodies to the viruses from clade 2.1.3.2 (A/Indo and A/Indonesia/NIHRD-12379/2012, A/Indo/12) in heterologous prime-boost groups primed with another 2.1.3.2. virus (A/Indo) were significantly higher than the responses to these two viruses in the homologous prime-boost group which only received 2 doses of A/Turkey (*p* < 0.05, Fig. 1a, b). Between the two heterologous prime-boost groups, the difference in antibody responses from high dose (group 2 at 7.5 µg HA/dose) versus standard dose (group 3 at 3.75 µg HA/dose) vaccination were not significant (Fig. 1a, b, *p* > 0.05).

Compared to antigenic characterization by primary infection ferret sera (Table 1), antibody responses in humans following vaccination with two doses of AS03_A_-adjuvanted antigens are broadened against viruses from heterologous clades, with lower reductions in titers and higher GMT ratios when comparing responses to heterologous clade versus those to the vaccine viruses (Fig. 1c, d). Although A(H5N8) A/GF/14 virus is antigenically the most distant from the vaccine viruses (256-fold reduction in HI titers by ferret antisera to A/Turkey (Table 1), surprisingly, following heterologous prime-boost vaccination, 34–40% of the subjects achieved post vaccination titer ≥40 and seroconversion by HI and 63–74% achieved the same criteria by MN to this A(H5N8) virus (Tables 3, 4 and Supplementary Table 1).

The US Food and Drug Administration (FDA) accelerated approval vaccine licensure criteria requires that based on HI titers, the lower limits of 95% CI for SPR are ≥70% and SCR are ≥40% for healthy adults.^14^ In the current analysis, the heterologous prime-boost group at 3.75 µg HA/dose (Group 3) achieved the best SPR rate, with lower limit of 95% CI of SPR ≥70% to 4 (by MN) and 3 (by HI) out of 8 viruses. The lowest antibody response was to the A(H5N8) virus from clade 2.3.4.4. It should be noted that subjects included in the current study were a subset that was randomly selected from strong responders to the vaccine components from the initial immunogenicity evaluation.^13^ As such, these SPR and SCR do not necessarily reflect the performance of the study groups from the original randomized trial. Regardless, these data still suggest that heterologous prime-boost strategy with AS03_A-_adjuvanted A(H5N1) A/Indo-A/Turkey vaccination, even administered at 18 months apart, may offer a broader range of cross-protection to drifted A(H5Nx) viruses.

Lastly, antibody responses to A(H5Nx) wild-type viruses from 8 clades measured by HI correlated well with neutralizing antibody responses measured by MN to each of the 8 viruses. The spearman correlation co-efficient (*r*) between HI and MN are *r* > 0.86 to 7 out of 8 viruses (*p* < 0.0001) and *r* > 0.58 for all 8 viruses (*p* < 0.0001) (Supplementary Fig. 2).

### Molecular and structural analysis of A(H5Nx) viruses

**Fig. 2:**
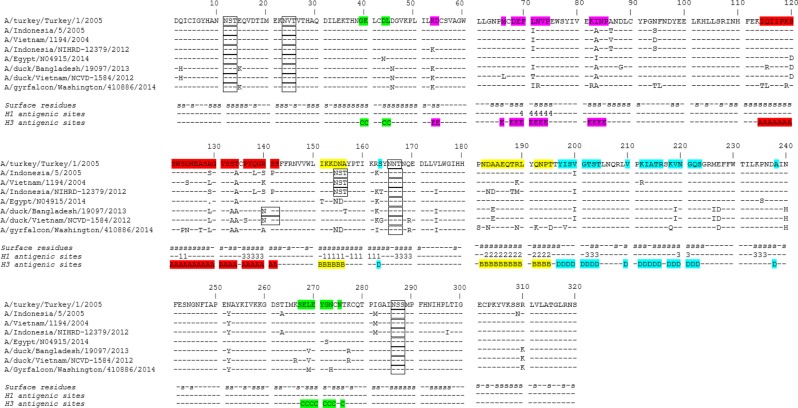
Structure-based sequence alignment of A(H5Nx) viruses used in the study. The locations equivalent to H1 and H3 antigenic sites are labeled with the antigenic site designation (Sa [1], Sb [2], Ca [3], or Cb [4] for H1 antigenic sites and A (red), B (yellow), C (green), D (aqua), or E (pink) for H3 antigenic sites. Glycosylation motifs (NXT/S) are boxed. Accession numbers: A/Indonesia/5/2005 (Genbank EF541394.1), A/turkey/Turkey/1/2005 (Genbank EF619980.1), A/Vietnam/1194/2004 (Genbank EF541402.1) A/Egypt/N04915/2014 (GISAID EPI_ISL_262572), A/duck/Bangladesh/19097/2013 (Genbank KF715205.1), A/gyrfalcon/Washington /410886/2014 (Genbank EPI569390). A/Indonesia/NIHRD-12379/2012 (GISAID EPI442759); and A/duck/Vietnam/NCVD-1584/2012 (EPI424977)

Both heterologous prime-boost vaccination groups mounted higher antibody responses (*p* < 0.01) than the homologous group against A/Indo/12, a virus from clade 2.1.3.2. Sequences of A(H5Nx) viruses were structurally aligned, positions equivalent to A(H3N2) and A(H1N1) antigenic sites on the HAs were analyzed (Fig. 2; Supplementary Table 2–5).^15^ Compared with A/Indo, A/Indo/12 has high sequence homology with 100%, 84%, 100%, 95%, and 93% amino acid sequence identity respectively in H3 equivalent HA antigenic sites A, B, C, D, and E^16,17^ (Fig. 2 and Supplementary Table 3), and ranged from 77 to 100% in amino acid sequence identity for positions equivalent to A(H1N1) antigenic sites (Fig. 2 and Supplementary Table 5), suggesting that the elevated responses to this virus in the heterologous prime-boost is likely due to the inclusion of a similar virus A/Indo (also from clade 2.1.3.2) as the priming antigen.

**Fig. 3:**
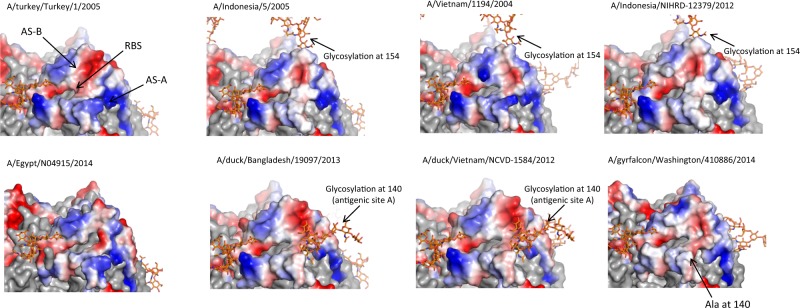
Structural models of hemagglutinin from A(H5Nx) viruses used in the study. Antigenic sites A and B (H3 equivalent) are colored as electrostatic surface, red indicates acidic and blue indicates basic. Sites A (AS-A) and B (AS-B) are the major antigenic sites surrounding the receptor binding site (RBS). Change in glycosylation side chain at position 154 and 140 are labeled

The addition of glycosylation sites on the HA of the viruses may also impact antigenicity. Compared to vaccine viruses A/Turkey and A/Indo, two viruses from clade 2.3.2.1: A/duck/Vietnam/NCVD-1584/2012 (2.3.2.1c) and A/duck/Bangladesh/19097/2013 (2.3.2.1a) both have an additional glycosylation site at position 140 of the HA (antigenic site A, Figs 2, 3). Both viruses have moderate levels of cross-reactivity and GMT ratio (0.30–0.41 by HI, 0.12–0.19 by MN) to the booster vaccine virus A/Turkey (Fig. 1c, d). In addition, A/Indo, A/Indo/12 and A/Vietnam/1194/2004 (A/Vietnam) share a glycosylation site at position 154 (antigenic site B, Figs 2, 3) of HA that may affect the immune response to antigenic site B.

A(H5N8) virus (A/GF/14) has the lowest amino acid homology in antigenic sites compared to the vaccine viruses. It only has 64, 68% sequence identity compared to A/Turkey, and 61, 68% sequence identity compared to A/Indo in antigenic sites A and B, respectively (H3 equivalent antigenic sites) (Supplementary Tables 2, 3). Moreover, A/GF/14 has a hydrophobic residue alanine (Ala) at position 140, whereas other viruses all have a charged hydrophilic residue at this exposed position on the HA (Figs 2, 3). Taken together, these molecular and structural features of the A/GF/14 virus likely contributed to the lowest cross-clade antibody responses (Tables 2, 3) and GMT ratio to booster vaccine A/Turkey (0.12–0.16 by HI and 0.09–0.12 by MN across all three groups, Fig. 1c, d) detected to this virus following either homologous or heterologous prime-boost vaccination with A/Turkey and A/Indo.

## Discussion

Identifying effective and efficient vaccination and stockpile strategies against a potential influenza pandemic is an essential component of the influenza pandemic preparedness. A prime-boost vaccination utilizing existing stockpiled vaccines in combination with newly developed vaccines containing a CVV antigenically well-matched with the pandemic virus may offer a strategy to broaden the protective efficacy of the vaccines and ease the surge in demand for vaccine doses in the event of a rapid onset of the pandemic. Pre-pandemic stockpiled vaccines can be used to prime the high-risk populations either at the early stage of a pandemic (pandemic scenario), or in advance (pre-pandemic scenario), followed by a boosting dose of vaccine that is well-matched with the pandemic strain when it becomes available. In addition, prior to the widespread availability of a well-matched pandemic vaccine, a prime-boost strategy solely based on the existing antigenically related stockpiled vaccines that offer partial protection against pandemic strains can also be of value. Here, we demonstrated that priming with a clade 2.1.3.2 antigen (A/Indo) followed by one booster dose of a clade 2.2.1 antigen (A/Turkey) broadened the cross-clade antibody responses to several antigenically drifted variants from 6 heterologous clades, including an antigenically distant A(H5N8) virus^18,19^ without compromising the antibody responses to the booster vaccine (A/Turkey).

HPAI A(H5N1) viruses have exhibited increasing genetic and antigenic diversity since their re-emergence in 2003. No viruses from one single H5 clade can serve as a vaccine that provides sufficient protection against all H5 viruses from heterologous clades. To-date, it is still impossible to predict which H5 viruses may gain efficient and sustained human-to-human transmission and cause the next influenza pandemic. Results from the current study, together with others,^11,20^ suggest that a heterologous prime-boost vaccination using A(H5N1) vaccine antigens with different antigenic properties may provide broader protection against novel strains by inducing more cross-reactive responses. In addition, the inclusion of oil-in-water emulsion-based adjuvants such as AS03_A_ and MF59 may further broaden antibody responses.

The design of a prime-boost antigen combination that can afford optimal coverage to emerging strains remains to be explored. Studies using primary infection sera generated in animals suggested that genetic distance based on the whole HA sequences do not always correlate with antigenic distance.^21^ Residues on the viral hemagglutinin protein that are around the receptor binding sites, key antigenic sites and those that alter glycosylation are more likely to have bigger impact on altering virus antigenicity.^21–23^ Furthermore, immune responses in humans following A(H5N1) vaccination can also be confounded by the use of potent oil-in-water adjuvants (for example stockpiled AS03_A_ and MF59),^24–26^ multiple doses, and the complex immune exposure history to seasonal influenza viruses. Compared to antigenic characterization using post-infection ferret antisera, antibody responses in humans following A(H5N1) vaccination are broadened against viruses from heterologous clades, with less fold-reductions in titers and higher GMT ratios to antigenically drifted viruses versus those to the vaccine viruses, even among the current study participants that had no pre-vaccination titers to A(H5N1) (Fig. 1c, d). Others also reported that a subset of the population have pre-existing antibodies to A(H5N1) viruses likely due to past exposure to seasonal viruses, and may influence their responses following A(H5N1) vaccination.^27,28^

In the current study, an A/Indo-A/Turkey prime-boost with AS03_A_ adjuvant elicited broad cross-clade antibody responses against multiple heterologous viruses. In subjects that were primed with an older antigen from clade 2.1.3.2 (A/Indo), a single booster dose with a clade 2.2.1 virus resulted in significantly higher antibody responses to a newer virus from the same priming clade (A/Indo/12) than those subjects that were not primed with A/Indo, suggesting a strong recall response to those shared epitopes between the older and newer clade 2.1.3.2 viruses in the heterologous prime-boost groups. Moreover, the addition of glycans on critical antigenic sites of the viruses may shield epitopes and alter antigenicity of the viruses. We hypothesize that an additional glycosylation site at position 140 in the antigenic site A of the two viruses from clade 2.3.2.1c and 2.3.2.1a may contribute to the moderate level of cross-reactivity detected to these two viruses following vaccination with A/Indo and A/Turkey, which both lack glycosylation site at this position. Likewise, a shared glycosylation site at position 154 of the HA on antigenic site B between A/Vietnam and A/Indo may affect immune responses to A/Vietnam following A/Indo priming. Additional studies are warranted to further identify structural epitopes that may influence the breadth of the antibody responses from A(H5N1) vaccination.

Surprisingly, despite the large antigenic, genetic and structural differences between the A(H5N8) virus and the two vaccine viruses, 63–74% of subjects in the heterologous groups still achieved post vaccination titers ≥40 and seroconversion by MN to A/GF/14, a recently emerged clade 2.3.4.4 virus that caused multiple outbreaks in domestic poultry and wild birds in the U.S. More interestingly, the antibody responses detected against A(H5N8) virus with A/Indo-A/Turkey prime-boost in the current study are superior than those detected from another heterologous prime-boost regimen we evaluated in a similar age cohort in adults. Subjects in the previous study that were primed with unadjuvanted A/VN/1194/2004 (clade 1) and boosted by a MF-59 adjuvanted clade 2.3.4.4. virus (A/Anhui/1/2005) administered 21 months later mounted little neutralizing antibody responses to the same A(H5N8) virus.^11^ Other studies also suggested that compared with non-adjuvanted antigen priming, priming with AS03_A_-adjuvanted vaccines can promote stronger memory responses upon boosting with heterologous strains.^27^ The differences in the cross-clade response against the antigenically distant A(H5N8) virus are likely due to the difference in antigen and adjuvant prime-boost combinations used in these studies. It is also to be noted that even in the heterologous prime-boost groups, licensure criteria was only achieved for some but not all of the antigenically drifted A(H5Nx) viruses.

A vaccination regimen that can induce long term immunity and provide protection to potential emerging pandemic strains is highly desirable for pandemic preparedness.^29^ In a naïve population, two doses of A(H5N1) vaccination are needed to induce sufficient antibody responses to provide protection. Here, we demonstrated that in a primed cohort, a single booster dose administered 18 months after the original priming offered rapid (10 days post-boost) and broad coverage to a panel of genetically and antigenically diverse A(H5Nx) viruses, suggesting a strong recall of immune memory and quick expansion of the long-lived memory B cells and plasma cells capable of differientating into plasmablasts and secreting HI and neutralizing antibodies, even after 18 months post-priming.^29^ The cross-clade heterologous antibody responses were detected by both HI and neutralizing assays with good correlation in titers. Moreover, there is no significant difference between the high and low HA dose heterologous prime-boost groups, consistent with previous studies that demonstrated the role of AS03_A_ on antigen sparing.^9^ Pandemics can occur rapidly, even in the matter of weeks after emergence.^30^ Our study only evaluated prime-boost schedule that is 12–18 months. In a pandemic setting, the relevance of heterologous prime-boost to broaden protection when the booster dose is administered at a short interval after the prime (e.g., 14–28 days) remains to be understood. Nevertheless, the long term booster schedule demonstrated in the current study offers flexibility in pre-pandemic planning. The quick rise and broad responses following a single booster dose of vaccine are encouraging. Surprisingly, even the homologous prime-boost group induced cross-reactive antibody responses to several A(H5Nx) drift variants, this could be especially valuable for countries that do not hold stockpiles of vaccines against multiple A(H5Nx) viruses.

Our study has several limitations, first, we could not include all A(H5Nx) viruses that caused recent human cases and outbreaks, as A(H5Nx) viruses continue to evolve, further studies may be needed when new antigenic variants emerge; second, our analysis is only focused on the strong responders from the immunogenicity evaluation of the original study, those that initially did not respond to the vaccine viruses would likely have little or no cross-reactive antibody responses to A(H5Nx) antigenic variants; Third, the two dose of homologous vaccination were given at 6 months interval, whereas the two doses of heterologous vaccination were given at 18 months interval, this difference in vaccination intervals may impact the comparisons.

In conclusion, this study demonstrated that heterologous prime-boost with adjuvanted stockpiled A/Indo and A/Turkey antigens can induce broad and robust cross-clade antibody responses to recent emerging strains. Our results also suggest that the levels of cross-clade antibody responses against novel viruses vary, depending on the antigenic distance from the vaccine viruses. The use of potent adjuvants likely broadens the responses. The molecular, structural and antigenic determinants of A(H5Nx) viruses that can drive the breadth of the vaccine responses following a prime-boost strategy, and the mechanism of boosting of immune memory in humans against novel A(H5Nx) viruses, must be better understood in order to design the most effective vaccination strategies utilizing stockpiled vaccines against novel viruses that could cause an influenza pandemic.

## Methods

### Clinical study and human sera

A(H5N1) vaccine sera were provided by GSK Vaccines. Stored sera were collected from healthy adults (≥18 years) enrolled in a GSK’s A(H5N1) vaccine trial (Clinical Trials.gov: NCT00719043).^13^ The original trial was a randomized, observer-blind, placebo controlled study including 7 study arms.^13^ Sera from subjects in three study arms (Table 2) who received AS03_A_ (adjuvant system 03 containing 11.8 mg α-tocopherol and squalene in an oil-in-water emulsion) with homologous prime-boost (group 1), and heterologous prime-boost (group 2 & 3), were included in the current study.

All subjects received 3 intramuscular injections (2 vaccines and 1 placebo) during the course of 18 months (Table 2). Subjects in the homologous prime-boost group (group 1) received two doses of AS03_A_ adjuvanted A/Turkey inactivated split virus antigen at 3.75 µg HA per dose (standard dose with AS03_A_ adjuvant), administered 12 months apart. Subjects from the heterologous prime-boost groups (groups 2 and 3) were primed with AS03_A_-adjuvanted A/Indo inactivated split virus antigen at day 0, and boosted with AS03_A_-adjuvanted inactivated split virus antigen A/Turkey 18 months later. Subjects from the two heterologous prime-boost groups (A/Indo-A/Turkey) differed by the vaccine antigen doses received: they either received 7.5 µg (high dose, group 2) or 3.75 µg HA antigen per dose (low dose, group 3).

The subjects evaluated in the current study are a subset of strong responders that achieved seroconversion by HI from the original immunogenicity evaluation of the clinical trial, which represents 42.9% of the study participants in the homologous group that received 2 doses of A/Turkey (group 1), 87.5% of the participants from the heterologous group that received A/Indo-A/Turkey at 7.5ug HA per dose (group 2), and 84.1% of the participants from the heterologous prime-boost group that received A/Indo-A/Turkey at 3.75 µg HA per dose of vaccines (group 3).^13^ Thirty-five subjects were randomly selected from strong responders for each of the 3 study groups that received either homologous or heterologous prime-boost A(H5N1) vaccination.

Paired sera collected at day 0 (pre-vaccination), and day 559 (approximately 10 days post- last dose) were analyzed. All subjects included in the current study gave consent for the testing of stored sera. Written informed consent were obtained from all participants. The use of sera was approved by the Centers for Disease Control and Prevention, National Centers for Immunization and Respiratory Diseases Human Research Determination Review.

### Study viruses

A total of 8 wild-type HPAI A(H5Nx) viruses from 6 genetic clades were used in the study, including the wild-type version of the vaccine strains and a panel of HPAI A(H5Nx) viruses from 6 genetic clades: A/Turkey (clade 2.2.1), A/Indo (clade 2.1.3.2), A/Vietnam/1194/2004 (clade 1, A/Vietnam), A/Indonesia/NIHRD-12379/2012 (clade 2.1.3.2, A/Indo/12), A/Egypt/N04915/2014 (clade 2.2.1), A/duck/Bangladesh/19097/2013 (clade 2.3.2.1a), A/duck/Vietnam/NCVD-1584/2012 (clade 2.3.2.1c), and A/gyrfalcon/Washington/410886/2014 (clade 2.3.4.4, A/GF/14). Viruses were propagated in the allantoic cavity of embryonated hen’s eggs at 37 °C for approximately 24 h. All research with A(H5N1) and A(H5N8) HPAI viruses was conducted in Biosafety level 3 Enhanced facilities.

### Serological assays

Sera were tested by hemagglutination inhibition (HI) and microneutralization (MN) assays. All assays were conducted in Biosafety level 3 Enhanced (BSL3E) laboratories following select agent requirements defined by US Department of Agriculture and the Select Agent Program.

### HI assays using horse erythrocytes

A modified HI assay using horse erythrocytes was performed to measure HI antibody responses.^11^ Human sera were heat inactivated, tested for non-specific agglutinins and adsorbed with packed horse erythrocytes as needed. All sera were then treated with receptor destroying enzyme (RDE) at 37 °C for 18–20 h, followed by heat inactivation prior to HI assays. Sera were two-fold serially diluted and incubated with 4 hemagglutination units (4HAU)/25 µl of virus for 30 min. Horse erythrocytes were added to the wells and incubated with virus and sera mixture at room temperature. Hemagglutination was read after 60 min. HI titer was defined as the reciprocals of the last dilutions of serum that completely inhibited hemagglutination. Antibody titers less than 10 (initial sera dilution) were reported as 5 for calculation purposes.

### Microneutralization assays (MN)

MN assays were performed to measure neutralizing antibody responses.^31^ Human sera were heat inactivated at 56 °C for 30 min. Two-fold serial diluted sera were incubated with 100 tissue culture infectious dose 50 (TCID_50_) of influenza viruses and incubated at 37 °C 5% CO_2_ for 1 h. The virus-sera mixture was used to infect 1.5 × 10^4^/well Madin-Darby Canine Kidney (MDCK) cells, and incubated for 18–20 h at 37 °C with 5% CO_2_. After cold acetone fixation, the presence of influenza viral protein was quantified by an ELISA using influenza A nucleoprotein (NP) monoclonal antibodies. MN titers were defined as the reciprocal of the highest dilutions of serum that gave 50% neutralization. Antibody titers less than 10 (initial sera dilution) were reported as 5 for calculation purposes.

### Molecular and structural analysis

A(H5Nx) models were generated by homology modeling.^32^ For structural-based sequence alignment, HA surface residues were determined based on A/Vietnam/1203/2004 (pdb: 2fk0),^33^ virus A(H5Nx) sequences were compared against A/Turkey. HA structural models were generated using PyMOL (Molecular Graphics System, Version 2.1.1 Schrödinger).

### Statistical analysis

Geometric mean titers (GMTs) of at least 2 replicates were reported as final titers for HI or MN. For each study group, GMT and their 95% confidence intervals (CI) were determined. “GMT ratio” is calculated as the GMT to the test virus divided by the GMT to the vaccine virus post vaccination for each study group. Seroprotection Rate (SPR) is the proportion of the subjects (%) that achieved a post-vaccination titer of ≥40. Seroconversion is defined as a 4-fold or greater rise from pre-vaccination to post-vaccination titer with post-vaccination titers of ≥40. Seroconversion rate (SCR) is the proportion of subjects (%) that achieved seroconversion per study group. Statistical comparison was analyzed using ANOVA and paired two-sided *t*-tests. Sample random selection and statistical analysis were performed using SAS^®^ 9.3 and GraphPad Prism 5.

### Reporting summary

Further information on experimental design is available in the Nature Research Reporting Summary linked to this article.

## Supplementary information

Supplementary Material

Reporting Summary

## Data Availability

The data supporting the findings of the study are available from the corresponding author upon reasonable request.

## Supplementary information

**Supplementary information** accompanies the paper on the *npj Vaccines* website (10.1038/s41541-019-0114-8).

## References

[1] WHO. Cumulative number of confirmed human cases for avian influenza A(H5N1) reported to WHO, 2003–2018, https://www.who.int/influenza/human_animal_interface/H5N1_cumulative_table_archives/en/ (2018). Accessed Dec 2018.

[2] Cox NJ, Trock SC, Burke SA (2014). Pandemic preparedness and the Influenza Risk Assessment Tool (IRAT). Curr. Top. Microbiol. Immunol..

[3] Smith GJ, Donis RO (2015). Nomenclature updates resulting from the evolution of avian influenza A(H5) virus clades 2.1.3.2a, 2.2.1, and 2.3.4 during 2013–2014. Influenza Respir. Virus.

[4] Baz M, Luke CJ, Cheng X, Jin H, Subbarao K (2013). H5N1 vaccines in humans. Virus Res..

[5] Writing Committee of the Second World Health Organization Consultation on Clinical Aspects of Human Infection with Avian Influenza, A. V. (2008). Update on avian influenza A (H5N1) virus infection in humans. N. Engl. J. Med..

[6] Uyeki TM (2009). Human infection with highly pathogenic avian influenza A (H5N1) virus: review of clinical issues. Clin. Infect. Dis..

[7] WHO. Antigenic and genetic characteristics of zoonotic influenza viruses and development of candidate vaccine viruses for pandemic preparedness, https://www.who.int/influenza/vaccines/virus/characteristics_virus_vaccines/en/ (2019). Accessed Dec 2018.

[8] Treanor JJ, Campbell JD, Zangwill KM, Rowe T, Wolff M (2006). Safety and immunogenicity of an inactivated subvirion influenza A (H5N1) vaccine. N. Engl. J. Med..

[9] Leroux-Roels I (2007). Antigen sparing and cross-reactive immunity with an adjuvanted rH5N1 prototype pandemic influenza vaccine: a randomised controlled trial. Lancet.

[10] Jennings LC, Monto AS, Chan PK, Szucs TD, Nicholson KG (2008). Stockpiling prepandemic influenza vaccines: a new cornerstone of pandemic preparedness plans. Lancet Infect. Dis..

[11] Levine MZ (2017). Cross-reactive antibody responses to novel H5Nx Influenza viruses following homologous and heterologous prime-boost vaccination with a prepandemic stockpiled A(H5N1) vaccine in humans. J. Infect. Dis..

[12] Jhung MA, Nelson DI (2015). Outbreaks of avian influenza A (H5N2), (H5N8), and (H5N1) among birds—United States, December 2014–January 2015. Morb. Mortal. Wkly Rep..

[13] Langley JM (2015). Immunogenicity of heterologous H5N1 influenza booster vaccination 6 or 18 months after primary vaccination in adults: a randomized controlled clinical trial. Vaccine.

[14] Center for Biologics Evaluation and Research, FDA, US Department of Health and Human Services. Guidance for Industry: clinical data needed to support the licensure of pandemic Influenza vaccines, https://www.fda.gov/files/vaccines,%20blood%20&%20biologics/published/Guidance-for-Industry-Clinical-Data-Needed-to-Support-the-Licensure-of-Pandemic-Influenza-Vaccines.pdf (2007). Accessed Dec 2018.

[15] Yang H (2016). Molecular characterizations of surface proteins hemagglutinin and neuraminidase from recent H5Nx Avian Influenza viruses. J. Virol..

[16] Wiley DC, Wilson IA, Skehel JJ (1981). Structural identification of the antibody-binding sites of Hong Kong influenza haemagglutinin and their involvement in antigenic variation. Nature.

[17] Stray SJ, Pittman LB (2012). Subtype- and antigenic site-specific differences in biophysical influences on evolution of influenza virus hemagglutinin. Virol. J..

[18] Pulit-Penaloza JA (2015). Pathogenesis and transmission of novel highly pathogenic Avian Influenza H5N2 and H5N8 viruses in ferrets and mice. J. Virol..

[19] Ip HS (2015). Novel Eurasian highly pathogenic avian influenza A H5 viruses in wild birds, Washington, USA, 2014. Emerg. Infect. Dis..

[20] Khurana S (2011). MF59 adjuvant enhances diversity and affinity of antibody-mediated immune response to pandemic influenza vaccines. Sci. Transl. Med..

[21] Peeters B (2017). Genetic versus antigenic differences among highly pathogenic H5N1 avian influenza A viruses: consequences for vaccine strain selection. Virology.

[22] Koel BF (2013). Substitutions near the receptor binding site determine major antigenic change during influenza virus evolution. Science.

[23] Duvvuri VR, Duvvuri B, Cuff WR, Wu GE, Wu J (2009). Role of positive selection pressure on the evolution of H5N1 hemagglutinin. Genom. Proteom. Bioinform..

[24] Banzhoff A (2009). MF59-adjuvanted H5N1 vaccine induces immunologic memory and heterotypic antibody responses in non-elderly and elderly adults. PLoS One.

[25] Garcon N, Vaughn DW, Didierlaurent AM (2012). Development and evaluation of AS03, an adjuvant system containing alpha-tocopherol and squalene in an oil-in-water emulsion. Exp. Rev. Vaccin..

[26] Moris P (2011). H5N1 influenza vaccine formulated with AS03 A induces strong cross-reactive and polyfunctional CD4 T-cell responses. J. Clin. Immunol..

[27] Leroux-Roels I (2010). Priming with AS03 A-adjuvanted H5N1 influenza vaccine improves the kinetics, magnitude and durability of the immune response after a heterologous booster vaccination: an open non-randomised extension of a double-blind randomised primary study. Vaccine.

[28] Nolan T (2008). Safety and immunogenicity of a prototype adjuvanted inactivated split-virus influenza A (H5N1) vaccine in infants and children. Vaccine.

[29] Tarlinton D, Good-Jacobson K (2013). Diversity among memory B cells: origin, consequences, and utility. Science.

[30] Wu JT (2010). The infection attack rate and severity of 2009 pandemic H1N1 influenza in Hong Kong. Clin. Infect. Dis..

[31] WHO. Manual for the laboratory diagnosis and virological surveillence of influenza (WHO Geneva, 2011), 59–62, https://www.who.int/influenza/gisrs_laboratory/manual_diagnosis_surveillance_influenza/en/. Accessed Dec 2018

[32] Waterhouse A (2018). SWISS-MODEL: homology modelling of protein structures and complexes. Nucleic Acids Res..

[33] Stevens J (2006). Structure and receptor specificity of the hemagglutinin from an H5N1 influenza virus. Science.

