# Supplementary material for: Heterologous prime-boost with A(H5N1) pandemic influenza vaccines induces broader cross-clade antibody responses than homologous prime-boost
Source: NPJ Vaccines. 2019 May 29;4:22. doi: 10.1038/s41541-019-0114-8 (PMC6541649; doi:10.1038/s41541-019-0114-8)
Supplement: Supplementary file 1 — Supplementary Material [file 41541_2019_114_MOESM1_ESM.pdf]

Supplementary Table 1. Cross-clade HI and neutralizing antibody seroconversion rates following prime-boost A(H5N1) vaccination

Study Groups	Influenza A Viruses	Subtype	HA Clade	N	HI	MN
					% seroconversion -SCR (95% CI)	% Seroconversion -SCR (95% CI)
Group 1 (homologous)	A/turkey/Turkey/1/2005	H5N1	2.2.1	35	86 (70-95)	94 (81-99)
	A/Indonesia/05/2005	H5N1	2.1.3.2	35	69 (51-83)	80 (63-92)
	A/Vietnam/1194/2004	H5N1	1	35	71 (54-85)	63 (45-79)
	A/Indonesia/NIHRD-12379/2012	H5N1	2.1.3.2	35	63 (45-79)	63 (45-79)
	A/Egypt/N04915/2014	H5N1	2.2.1	35	66 (48-81)	86 (70-95)
	A/duck/Bangladesh/19097/2013	H5N1	2.3.2.1a	35	66 (48-81)	71 (54-85)
	A/duck/Vietnam/NCVD-1584/2012	H5N1	2.3.2.1c	35	57 (39-74)	57 (39-74)
	A/gyrfalcon/Washington/410886/2014	H5N8	2.3.4.4	35	26 (12-43)	60 (42-76)
Group 2 (heterologous –high dose)	A/turkey/Turkey/1/2005	H5N1	2.2.1	35	83 (66-93)	97 (85-100)
	A/Indonesia/05/2005	H5N1	2.1.3.2	35	83 (66-93)	91 (77-98)
	A/Vietnam/1194/2004	H5N1	1	35	77 (60-90)	69 (51-83)
	A/Indonesia/NIHRD-12379/2012	H5N1	2.1.3.2	35	83 (66-93)	83 (66-93)
	A/Egypt/N04915/2014	H5N1	2.2.1	35	69 (51-83)	83 (66-93)
	A/duck/Bangladesh/19097/2013	H5N1	2.3.2.1a	35	80 (63-92)	80 (63-92)
	A/duck/Vietnam/NCVD-1584/2012	H5N1	2.3.2.1c	35	74 (57-88)	74 (57-88)
	A/gyrfalcon/Washington/410886/2014	H5N8	2.3.4.4	35	40 (24-58)	63 (45-79)
Group 3 (heterologous-low dose)	A/turkey/Turkey/1/2005	H5N1	2.2.1	35	91 (77-98)	97 (85-100)
	A/Indonesia/05/2005	H5N1	2.1.3.2	35	91 (77-98)	91 (77-98)
	A/Vietnam/1194/2004	H5N1	1	35	77 (60-90)	77 (60-90)
	A/Indonesia/NIHRD-12379/2012	H5N1	2.1.3.2	35	89 (73-97)	89 (73-97)
	A/Egypt/N04915/2014	H5N1	2.2.1	35	71 (54-85)	86 (70-95)
	A/duck/Bangladesh/19097/2013	H5N1	2.3.2.1a	35	74 (57-88)	80 (63-92)
	A/duck/Vietnam/NCVD-1584/2012	H5N1	2.3.2.1c	35	69 (51-83)	74 (57-88)
	A/gyrfalcon/Washington/410886/2014	H5N8	2.3.4.4	35	34 (19-52)	71 (54-85)

CI: confidence interval

Supplementary Table 2. Virus amino acid identity compared to A/turkey/Turkey/1/2005 based on H3 Antigenic sites.

Influenza A Viruses	% amino acid Identity to A/turkey/Turkey/1/2005 based on H3 Antigenic sites				
	Site A	Site B	Site C	Site D	Site E
A/turkey/Turkey/1/2005	100	100	100	100	100
A/Indonesia/05/2005	82	84	100	95	86
A/Vietnam/1194/2004	86	79	100	95	86
A/Indonesia/NIHRD-12379/2012	82	68	100	91	79
A/Egypt/N04915/2014	89	84	85	100	100
A/duck/Bangladesh/19097/2013	79	89	92	91	79
A/duck/Vietnam/NCVD-1584/2012	79	95	92	86	71
A/gyrfalcon/Washington/410886/2014	64	68	85	91	64

Supplementary Table 3. Virus amino acid identity compared to A/Indonesia/05/2005 based on H3 Antigenic sites.

Influenza A Viruses	% amino acid Identity to A/Indonesia/05/2005 based on H3 Antigenic Sites				
	Site A	Site B	Site C	Site D	Site E
A/Indonesia/05/2005	100	100	100	100	100
A/turkey/Turkey/1/2005	82	84	100	95	86
A/Vietnam/1194/2004	82	95	100	91	100
A/Indonesia/NIHRD-12379/2012	100	84	100	95	93
A/Egypt/N04915/2014	82	84	85	95	86
A/duck/Bangladesh/19097/2013	75	84	92	95	93
A/duck/Vietnam/NCVD-1584/2012	75	79	92	91	79
A/gyrfalcon/Washington/410886/2014	61	68	85	86	79

Supplementary Table 4. Virus amino acid identity compared to A/turkey/Turkey/1/2005 based on H1 Antigenic sites.

Influenza A Viruses	% amino acid Identity to A/turkey/Turkey/1/2005 based on H1 Antigenic sites			
	Ca	Cb	Sa	Sb
A/turkey/Turkey/1/2005	100	100	100	100
A/Indonesia/05/2005	88	83	69	100
A/Vietnam/1194/2004	94	83	69	92
A/Indonesia/NIHRD-12379/2012	88	83	62	77
A/Egypt/N04915/2014	94	100	77	100
A/duck/Bangladesh/19097/2013	94	83	85	92
A/duck/Vietnam/NCVD-1584/2012	82	83	85	92
A/gyrfalcon/Washington/410886/2014	88	67	62	69

Supplementary Table 5. Virus amino acid identity to A/Indonesia/05/2005 based on H1 Antigenic Sites

Influenza A Viruses	% amino acid Identity to A/Indonesia/05/2005 based on H1 Antigenic Sites			
	Ca	Cb	Sa	Sb
A/Indonesia/05/2005	100	100	100	100
A/turkey/Turkey/1/2005	88	83	69	100
A/Vietnam/1194/2004	88	100	85	92
A/Indonesia/NIHRD-12379/2012	100	100	92	77
A/Egypt/N04915/2014	82	83	85	100
A/duck/Bangladesh/19097/2013	88	100	85	92
A/duck/Vietnam/NCVD-1584/2012	76	83	69	92
A/gyrfalcon/Washington/410886/2014	82	83	62	69

Supplementary Figure 1

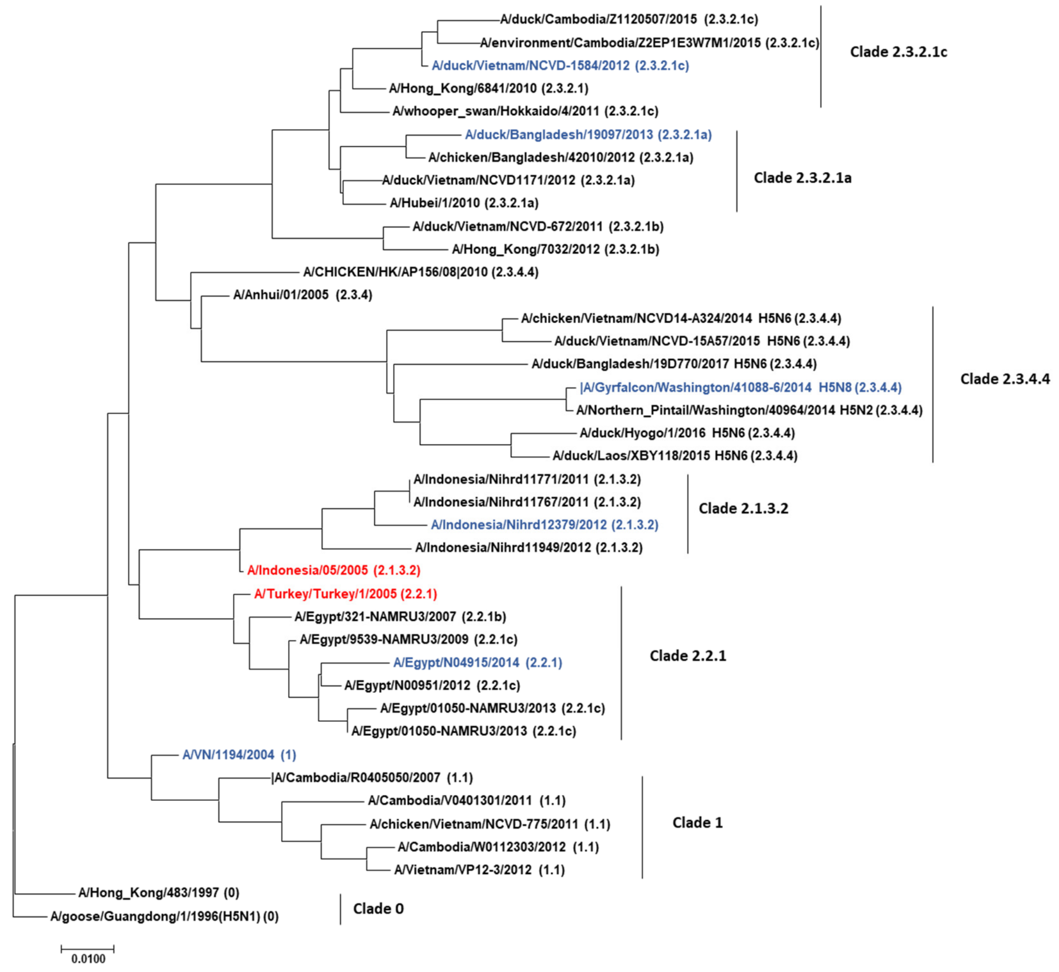

Supplementary Figure 1. Phylogenetic analysis of the A(H5Nx) viruses used in the study. Phylogenetic tree was constructed based on nucleotide sequences using maximum likelihood algorithm by Mega software 7.0.26. Vaccine viruses are highlighted in red, emerging viruses included in the study are highlighted in blue. Viruses analyzed are A(H5N1) unless indicated otherwise. Scale bar represents the average number of nucleotide substitutions per site.

Supplementary Figure 2

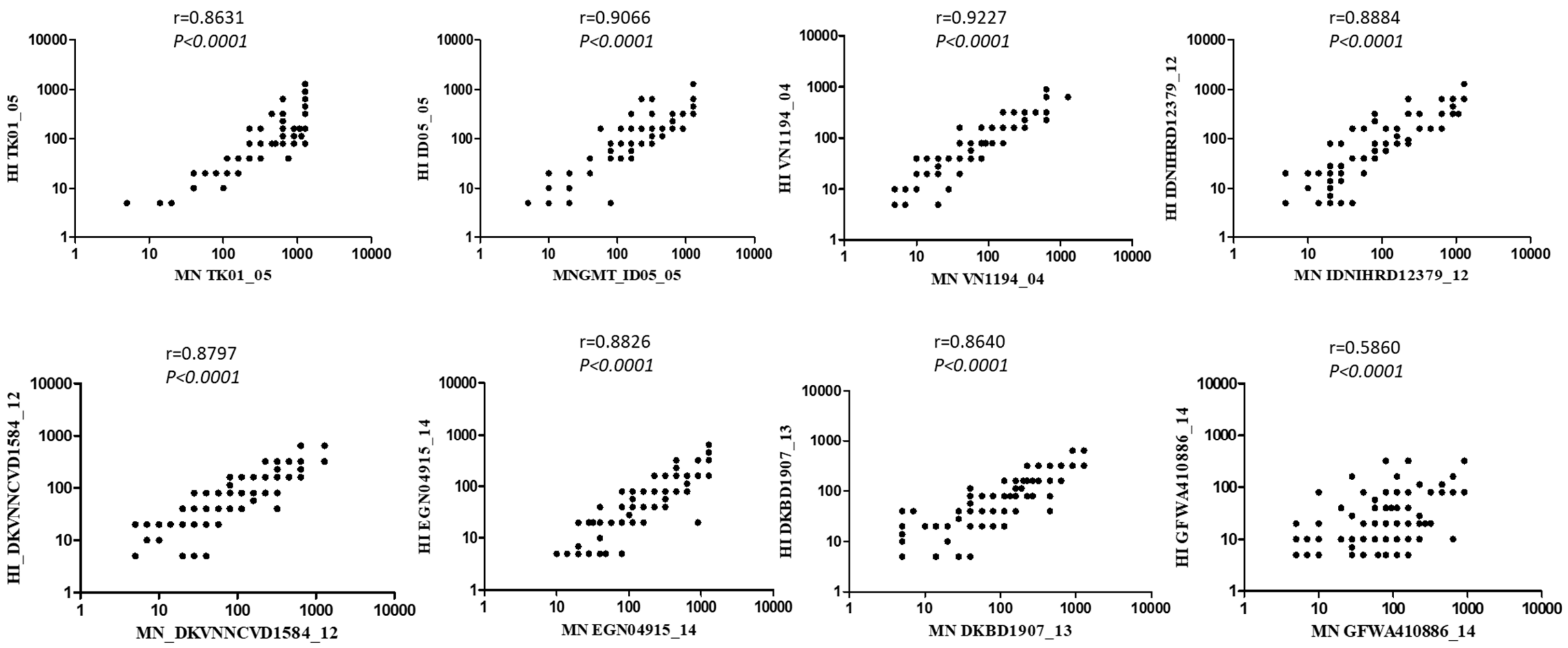

Supplementary Figure 2. Spearman correlation of MN and HI titers for each A(H5Nx) viruses post prime-boost vaccination. The correlation of MN versus HI titers for each virus was plotted.
VN1194_04 (A/Vietnam/1194/2004); ID05_05(A/Indonesia/5/2005); IDNIHRD12379 (Indonesia/NIHRD-12379/2012); TK01_05(A/turkey/Turkey/1/2005); EGN04915_14(A/Egypt/N04915/2014); DKBD19097_13(duck/Bangladesh/19097/2013); DKVNNCVD1584_12 (A/duck/Vietnam/NCVD-1584/2012); GFWA410886_14 (A/gyrfalcon/Washington/410886/2014)
